# Integrated analysis of single-cell RNA-seq and ATAC-seq in lens epithelial cells: Unveiling the role of ATF6 as a key transcription factor

**DOI:** 10.1016/j.gendis.2025.101610

**Published:** 2025-03-22

**Authors:** Huiping Lu, Xianyang Liu, Qian Zhou, Ruonan Li, Hangjia Zuo, Ke Hu, Meng Tian, Hong Wang, Jianqiao Li, Na Li, Shengping Hou

**Affiliations:** a The First Affiliated Hospital of Chongqing Medical University, Chongqing 400016, China; b Chongqing Key Laboratory of Ophthalmology, Chongqing 400016, China; c Chongqing Eye Institute, Chongqing 400016, China; d Department of Ophthalmology, Qilu Hospital, Shandong University, Jinan, Shandong 250062, China; e Department of Laboratory Medicine, Beijing Tongren Hospital, Capital Medical University, Beijing 100730, China; f Beijing Institute of Ophthalmology, Beijing Tongren Eye Center, Beijing Tongren Hospital, Capital Medical University, Beijing Ophthalmology & Visual Sciences Key Laboratory, Beijing 100730, China

**Keywords:** Activating transcription factor 6, Cataract, Epithelial-mesenchymal transition, Fiber cell, Lens epithelium, Single-cell sequencing

## Abstract

Lens epithelium, a fundamental biological structure pivotal for maintaining normal vision, can be disrupted, leading to the development of cataracts. The epithelial-mesenchymal transition has been proven to be the key factor of secondary cataract progression. However, the underlying mechanism of epithelial-mesenchymal transition in lens epithelial cells remains unclear. In this study, we conducted a comprehensive analysis and classification annotation of single-cell transcriptomic sequencing (scRNA-seq) data. This data was derived from fetal eye tissues of ages ranging from 9 to 23 weeks, sourced from our previously published research. Trajectory analysis showed a differentiation trend from epithelial cell to fiber cell. Furthermore, an integrative analysis of accessible-chromatin sequencing (scATAC-seq) and single-cell RNA sequencing (scRNA-seq) data revealed that the transcription factor ATF6 may play a pivotal role in maintaining the homeostasis of lens epithelial cells. Subsequent *in vitro* experiments revealed that inhibition of ATF6 could alleviate epithelial-mesenchymal fibrosis by reducing STAT3 phosphorylation. Collectively, our study presents an atlas of lens epithelial cell development at the single-cell resolution, uncovering evidence that heightened ATF6 activity could potentially promote epithelial-mesenchymal transition in lens epithelial cells.

## Introduction

The lens is a specialized transparent tissue composed of epithelial cells located in the anterior part of the lens and a mass of fibrous cells forming the remaining lens volume.^1^ Any alteration in the structure, organization, or function of these cells can lead to clouding of the transparent lens, resulting in the development of cataracts. Cataracts stand as the second leading cause of blindness globally, contributing to approximately 30% of blindness cases worldwide.^2^ In China, the incidence of cataract cases among individuals aged 45–89 years old has escalated from 50.75 million in 1990 to 111.74 million in 2015. Currently, apart from intraocular lens replacement, cataracts still lack effective treatment, and there is an urgent need for new therapeutic strategies for this condition.

Lens epithelial cells remain active throughout the life cycle, showing distinct physiological characteristics and functions at different developmental stages.^3^ In fetal development, these cells undergo differentiation into fiber cells through a process involving proliferation and differentiation. In post-maturity lens, epithelial cells contribute to maintaining lens homeostasis by engaging in activities such as material transport, synthesis, metabolism, and mitotic proliferation.^4^^,^^5^ This phase is susceptible to influences from both genetics and environmental factors. Any disturbance during this period can result in decreased lens transparency and cataract formation.^6^ Mounting evidence supports that epithelial-mesenchymal transition (EMT) of the lens is closely associated with the secondary cataract progression, however, the underlying mechanism remains unclear.

The advent of single-cell sequencing technology has profoundly revolutionized the field of cellular cognition, providing unprecedented opportunities for deciphering cell type heterogeneity and elucidating animal developmental processes.^7^^,^^8^ The application of this technology in the realm of eye development and the research of ophthalmic diseases is still in its early stages. The current literature in this area includes: i) the creation of retinal atlases^9^, ^10^, ^11^; ii) the exploration of the pathobiology of ocular diseases, including diabetic retinopathy, glaucoma, and age-related macular degeneration^12^^,^^13^; and iii) a limited number of studies related to the lens. Joshua R. Sanes et al constructed an anterior ganglion cell atlas of the human eye, in which they identified five lens cell subgroups with specific markers and delineated the expression of disease-associated genes across diverse cell populations.^14^ Employing single-cell sequencing technology, it is revealed that adult lens epithelial cells harbor an autophagy-prominent cell cluster situated in the central region of the lens capsule.^15^ Single-cell sequencing has been characterized in lens cells from adults, zebrafish, and chickens, but has rarely been reported in the human embryo. To date, however, there is a scarcity of research exploring the regulatory mechanisms governing lens development during fetal life using single-cell transcriptomic sequencing (scRNA-seq) technology.

In this study, we employed data analytics and classification annotations on scRNA-seq obtained from fetal eye tissue aged 9–23 weeks. Through this analysis, we identified a total of 1159 lens cells, accurately annotated as lens epithelial cells and fiber cells by adjusting the resolution. Furthermore, an integrative analysis of accessible-chromatin sequencing (scATAC-seq) and single-cell RNA sequencing (scRNA-seq) data has revealed that activating transcription factor 6 (ATF6) may play a pivotal role in maintaining the homeostasis of lens epithelial cells. Subsequent *in vitro* experiments revealed that ATF6 could facilitate the EMT in epithelial cells by orchestrating the regulatory dynamics of phosphorylated signal transducer and activator of transcription 3 (p-STAT3) expression. In summary, our findings identify a potential therapeutic target for the treatment of secondary cataracts.

## Materials and methods

### scRNA and scATAC sequencing data

The sample data used in our study was derived from our laboratory's article published in *Advanced Science* (DOI: 10.1002/advs.202206623) (GSE228370). Additionally, the collection of human embryonic and fetal ocular material was approved by the Ethics Committee of Chongqing Medical University's First Affiliated Hospital and was carried out in accordance with the approved guidelines (No. 2019-100-2). All donors provided signed informed consent and agreed to donate aborted fetuses to this study.

In our study, we focus explicitly on isolating and analyzing lens epithelial cells from the embryonic eye data. Subsequently, we conducted comprehensive analyses of scRNA-seq and scATAC-seq data from these cells (Tables S1 and S2).

### Cell culture and reagents

The human lens line SRA01/04 cell was purchased from Shanghai Fuheng Biotechnology Co., Ltd., and cultured in Dulbecco's modified Eagle's medium (DMEM, Gibco, USA) containing 10% fetal bovine serum in an incubator at 37 °C with 5% CO_2_. For the cell fibrosis model, SRA01/04 cells were treated with 10 ng/mL of transforming growth factor beta 1 (TGF-β1) (PeproTech, Suzhou, China) for 48 h. Ceapin-A7 was purchased from TargetMol Chemicals Inc. (MA, USA).

### Isolation of primary lens epithelial cells

The mice were decapitated and placed in 75% alcohol for disinfection and sterilization. The mice were dissected in a laminar flow hood to remove the eyeballs. The eyeballs were soaked for 10 min in a solution prepared by dissolving 2.5 g of gentamicin and 3 g of lincomycin in 10 mL of physiological saline solution. The eyeballs were cut open along the limbus, the lenses were removed, and a circumferential incision about 2 mm behind the equator of the posterior capsule was made. The anterior capsule and the equatorial capsule were carefully peeled off and completely removed using micro-toothless forceps. The removed capsules were immediately placed into a wide-mouth vial filled with 1 mL of DMEM culture medium containing 20% fetal bovine serum. Capsule scissors were used to cut the capsules into tissue pieces about 1 mm in diameter. The culture medium along with the tissue pieces was aspirated and placed into a culture dish, followed by incubation in a 5% CO_2_ incubator at 37 °C. After 24 h, 0.5 mL of culture medium was carefully added, followed by daily observation under a phase-contrast microscope. When cells were seen growing from the edges of the tissue pieces, 3 mL of culture medium was added to continue the culture.

### Western blot analysis

After three washes with phosphate buffer saline, cells were lysed using a radioimmunoprecipitation lysis buffer (Beyotime, Shanghai, China) with 1% protease inhibitor (Beyotime, Shanghai, China). The protein concentration of the sample was detected using the Bicinchoninic Acid Kit (Beyotime, Shanghai, China). Subsequently, the protein was separated by running gel and electro-imprinted onto a polyvinylidene fluoride membrane. Then, the membrane was blocked by Fast Blocking Western solution (Yeasen, Shanghai) and incubated with primary antibody at 4 °C overnight.^16^ Finally, the band was visualized by an enhanced chemiluminescence kit (KF8001, Affinity) and quantified with Image J. The primary antibodies used in this study were as follows: ATF6 (1:1000, Abcam), FN1 (1:1000, Proteintech), VIMENTIN (1:800, HuaBio), α-SMA (1:1500, Abcam), p-STAT3 (1:800, Abcam), STAT3 (1:800, Abcam), and β-actin(1:5000, Affinity).

### Immunofluorescence assay

SRA01/04 cells were seeded on slides and washed with phosphate buffer saline three times, then were fixed in 4% paraformaldehyde for approximately 15 min, and infiltrated with 1% Triton X-100 for 15 min, followed by three washes. The cells were then incubated with the primary antibodies at 4 °C for 16 h. After three washes, the cells were treated with corresponding secondary antibodies at room temperature for 1 h. The images were taken using the fluorescence microscope (Leica, Germany).

### scRNAseq analysis

The Seurat R package was used for scRNA-seq data processing. This package performs preprocessing and normalization steps, such as scaling gene expression across cell types and log transformation, to ensure data consistency. UMAP (uniform manifold approximation and projection)^17^ was then used for data downscaling and cluster identification. This technique can effectively reduce dimensionality and identify cell clusters based on their similarity. To integrate different datasets in Seurat4,^18^ we performed hierarchical clustering to identify the closest similarities among datasets. Canonical correlation analysis was used to align the datasets, and dynamic time warping was performed to ensure alignment accuracy. The integrated datasets were then reduced into a lower dimensional space using dimensional reduction. After performing all these steps, the datasets were combined using mutual nearest neighbors to create anchors and correction vectors were constructed along these anchors. These correction vectors were subsequently utilized to transpose the data from the second dataset into a compatible space for alignment with the first dataset. Clustering resolution for all datasets was set to match known cell type markers. If a cluster expressed markers from multiple groups, it was identified as a hybrid of multiple cell types. These cell types were then assigned to the appropriate clusters in the integrated objects. The final result was a comprehensive overview of the cell types across multiple datasets, including the percentage of cells that expressed a given gene within each cluster.

### Pseudotime analyses

To explore the process of lens cell development, we used monocel3^19^ to construct pseudotime developmental trajectories of four subtypes of lens epithelial cells, transitional cells, lens fiber cells, and other cell types obtained by Seurat analysis. The root node was set according to cells with high expression of PAX6. Using the Graph-autocorrelation analysis method, we identified genes that varied over a trajectory or between different clusters.

### Cell cycle analysis

We determine a score for each cell, based on its level of expression of G2/M and S phase markers. This score was determined using the CellCycleScoring function within Seurat 4. This function stores the S and G2/M scores in object metadata, along with the predicted classification of each cell as either G2M, S, or G1 phase.^20^

### Transcription factor activity inference

The Dorothea tool (https://saezlab.github.io/dorothea/) was employed to infer the expression levels of transcription factors and their direct target genes across a range of severity groups. This tool implements a method that uses mRNA expression data to construct transcription factor regulons, which represent the transcriptional state of the transcription factor and its direct targets. We used the “dorothea_regulon human” wrapper function from the “Dorothea” library version 0.99.10 to create transcription factor regulons. We selected two high-confidence transcription factors, “A” and “B”, for further analysis. The Viper scores of the transcription factor regulons were calculated and added to the integrated Seurat object as the “Dorothea” slot.

To enable comparison of transcription factor activity scores, we calculated the mean and standard deviation values of the scaled Viper scores for each severity group. We then ranked the transcription factors according to the variance of their Viper scores. The top 50 highly variable scores per severity group were selected for visualization.

### Function analysis

The “Metascape” (https://metascape.org/gp/index.html)^26^ software was utilized to investigate and visualize the functional profiles (gene ontology/GO and REACTOME) of genes and gene clusters online. The pathway/process enrichment analysis employed the standard accumulative hypergeometric statistical test to identify ontology terms with a significant presence of input genes.

### ATAC-seq data analysis

We utilized the R software package “ArchR” to analyze scATAC-seq data initially.^21^ We integrated scRNA and scATAC data to identify and predict the chromatin open regions in lens epithelial cells and lens fiber cells. The peak calling procedure was accomplished using the MACS2 algorithm, which identified areas of sequence tag enrichment. Furthermore, we utilized the ATF6 consistency sequence to traverse the open chromatin regions of the lens epithelial cells, capturing potential binding regions or genes of ATF6 in lens epithelial cells. In this program, we used the online analysis software FIMO (https://meme-suite.org/meme/tools/fimo).^22^

### Statistics analysis

All experiments performed in this study were at least three independent replicates. The data were presented as mean ± standard deviation and statistical analyses were performed utilizing SPSS Statistics 27 (IBM Corp., USA). Figures were generated using Prism 9.0 (GraphPad, USA).

## Results

### Identification of distinct clusters of lens epithelial cells and lens fiber cells

**Figure 1:**
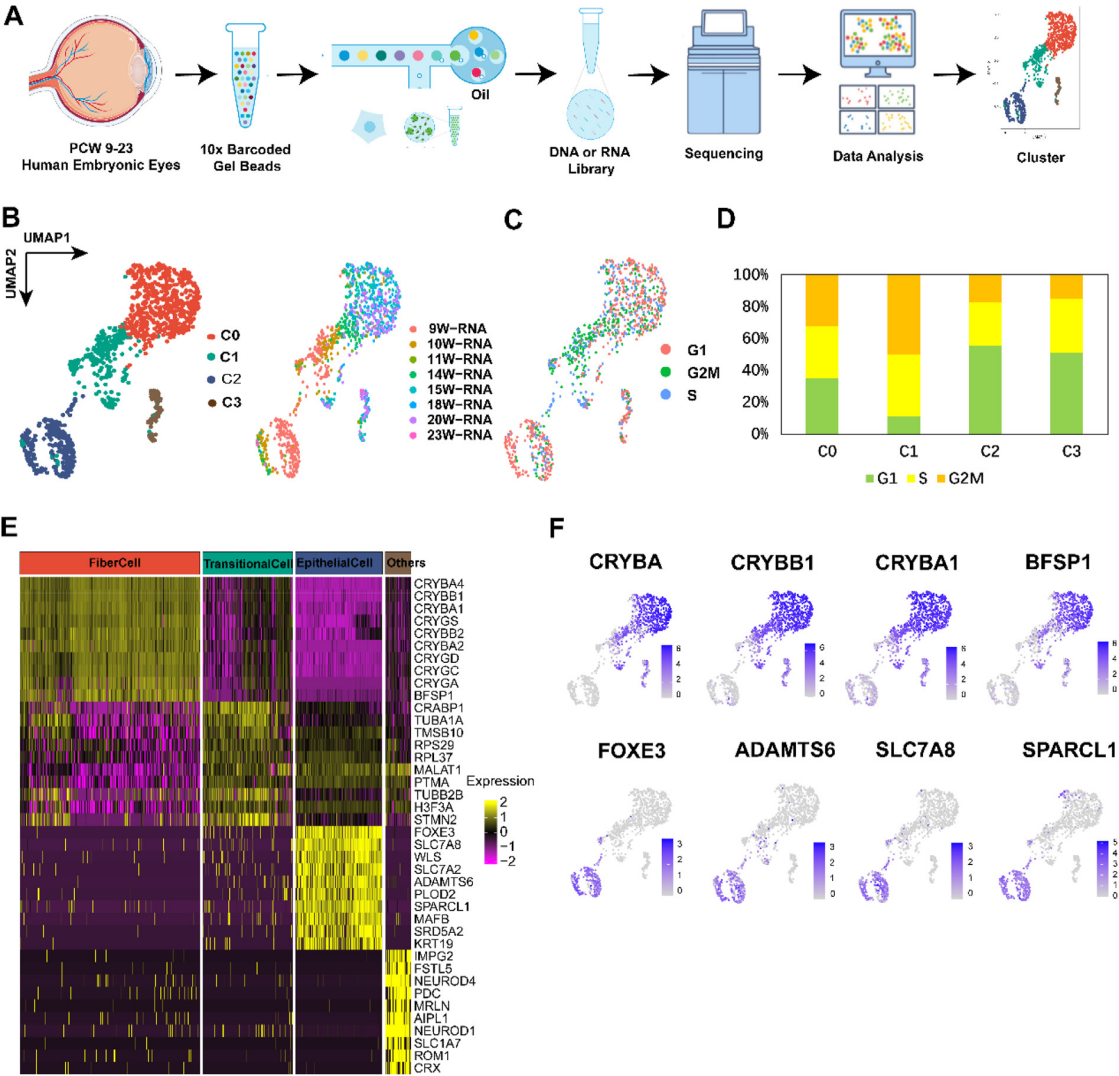
Single-cell RNA sequencing (scRNA-seq) identified four clusters of human embryonic eye lens cell clusters. **(A)** Schematic diagram of the human embryonic eyes design. **(B)** The uniform manifold approximation and projection (UMAP) algorithm was used to group the fetal lens scRNA-seq data into four clusters, each representing a unique combination of cell type and age. **(C)** UMAP embedding of the fetal lens scRNA-seq dataset, with individual cells colored by cell cycle. **(D)** The distribution of cell cycles in each cluster. **(E)** Within each fetal lens cluster, the top 10 most significant differences were observed in four distinct groups, namely lens fiber cells (FiberCell), transitional cells (TransitionalCell), lens epithelial cells (EpithelialCell), and unknown cluster (Others). **(F)** Marker gene expression distribution in lens epithelial cells and lens fiber cells.

To comprehensively characterize gene expression between lens epithelial cells and fiber cells, we employed the 10× Genomics platform to analyze the data of 79,111 single-cell transcriptomes (Table S1) and 69,014 single-cell epigenomes from 9- to 23-week-old human fetal eyes (Table S2). The data was sourced from our previously published research (GEO228370).^23^ Using the single-cell transcriptomic data analytics software Seurat 4.0,^18^ we identified lens cells based on the expression of lens-specific protein genes (CRYGC, BFSP1/2, and LIM2). 1159 lens cells were identified in human fetal eyes (Fig. 1A). We performed 0.15-resolution clustering analysis on this group of lens cells and generated four subgroups as follows: cluster 0 → fiber cells; cluster 1 → transitional cells; cluster 2 → epithelial cells; cluster 3 → others (Fig. 1B). Cell labeling based on fetal developmental stages revealed that most cells in cluster 2 and cluster 1 were from younger fetal stages, while cluster 0 and cluster 3 were skewed towards older fetal ages (Fig. 1C). Subsequently, we evaluated each cell by quantifying the expression levels of G2M or S phase marker genes. The findings indicated that most cells in cluster 2 transitioned into the G1 phase, while cells in cluster 1 predominantly progressed to the G2M phase (Fig. 1D). Interestingly, lens epithelial cells were mainly distributed at week 9, 10, and 11 in the first trimester, and then the proportion of lens fiber cells was expanded, which was significantly higher than that of epithelial cells (Fig. S1A).

To identify distinctive markers for the aforementioned four clusters, we executed specialized algorithms designed for characteristic gene expression analysis. The heat map illustrated the top 10 specific expression genes of each cluster (Fig. 1E). Specifically, cluster 0 expressed lens fiber genes (CRYBA4, CRYBB, CRYBA1, and BFSP1), cluster 1 displayed expression of cell membrane function genes, cluster 2 demonstrated expression of lens epithelial cell genes (forkhead box E3/FOXE3),^24^ and cluster 3 showed expression of light-sensitive genes (Fig. 1F).

We have conducted a comparative analysis of crucial transcription factor data obtained from lens epithelial cell and lens fiber cell cluster marker genes as described from adults,^25^ zebrafish,^26^ and chickens,^27^ alongside our findings. In our study, we noticed consistent expression patterns in adult lens studies, with C8orf8 and ADAMTSL4 being expressed in the lens epithelial cells, and CD24 being expressed in the lens fiber cells. Our data analysis, together with our review of the related chicken lens development article, indicates that markers of lens epithelial cells and lens fiber cells display consistent levels of expression, while markers of the intermediate process do not exhibit consistent expression levels, and high expression levels of these markers are not observed in our datasets. Key transcription factors that are highly expressed in lens epithelial cells include lens epithelial cell-specific overexpression markers FOXE3, HES4, SLC16A1, and TINAGL1. In our study, three key transcription factors in zebrafish lens development, APX1, PITX3, and CELFI were highly expressed in the lens epithelial cell cluster. Within our lens epithelial cells, we observed a high level of expression of progenitor cell markers HES5, PAX6, and SOX2 (Fig. S1B). Additionally, we mapped the expression of fetal lens marker genes (Fig. S1C) and found that the trends were consistent with those observed in adult lenses by snRNA-seq.^14^

### Lens epithelial cells are identified to undergo conversion into fiber cells through trajectory analysis

**Figure 2:**
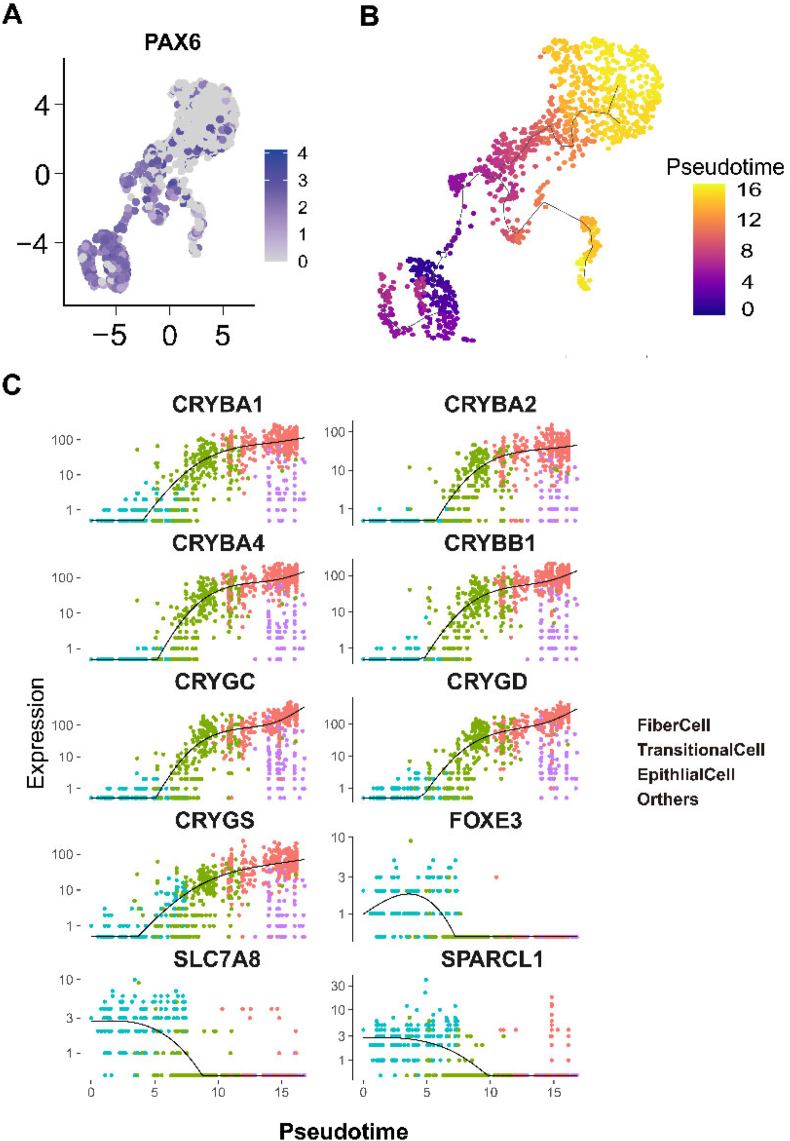
Pseudotime trajectory analysis of the fetal lens. **(A)** PAX6 was classified as lens epithelial cells due to the root of its pseudotime trajectory. **(B)** The pseudotime trajectory analysis revealed the direction of cell differentiation from lens epithelial cells to lens fiber cells. **(C)** The differentiation process driver genes identified included CRYBA1/2/4, CRYBB1, CRYGC/D/S, FOXE3, SLC7A8, and SPARCL1.

To explore the developmental trajectories of lens subsets in the fetal eye, we used monocle3 for pseudotime trajectory analysis. The root node for this analysis was defined based on the expression of the stem cell property marker gene PAX6.^28^ Given the high expression of PAX6 in cluster 2 (lens epithelial cells), it was chosen as the root node (Fig. 2A). Subsequently, these cells were subjected to gene expression analysis, and the results aligned with our initial hypothesis. The developmental trajectory was unfolded from cluster 2 (lens epithelium) to cluster 1 (intermediate state), and finally to cluster 0 (lens fiber) (Fig. 2B). Consequently, two categories of developmental driver genes were identified, one with gradual overexpression (*i.e.*, CRYBA1/2, CRYBA4, CRYBB1, CRYGC, CRYGD, and CRYGS; associated with cell fibrin expression) and the other with gradual decrease (*i.e.*, FOXE3, solute carrier family 7 member 8/SLC7A8, and SPARC like 1/SPARCL1; known for their role in cell cycle and cell pressure) (Fig. 2C). In summary, these findings suggest that lens epithelial cells transform into lens fiber cells with increasing developmental time during the fetal period.

### ATF6 is identified as a crucial transcription factor for lens epithelial cell homeostasis

**Figure 3:**
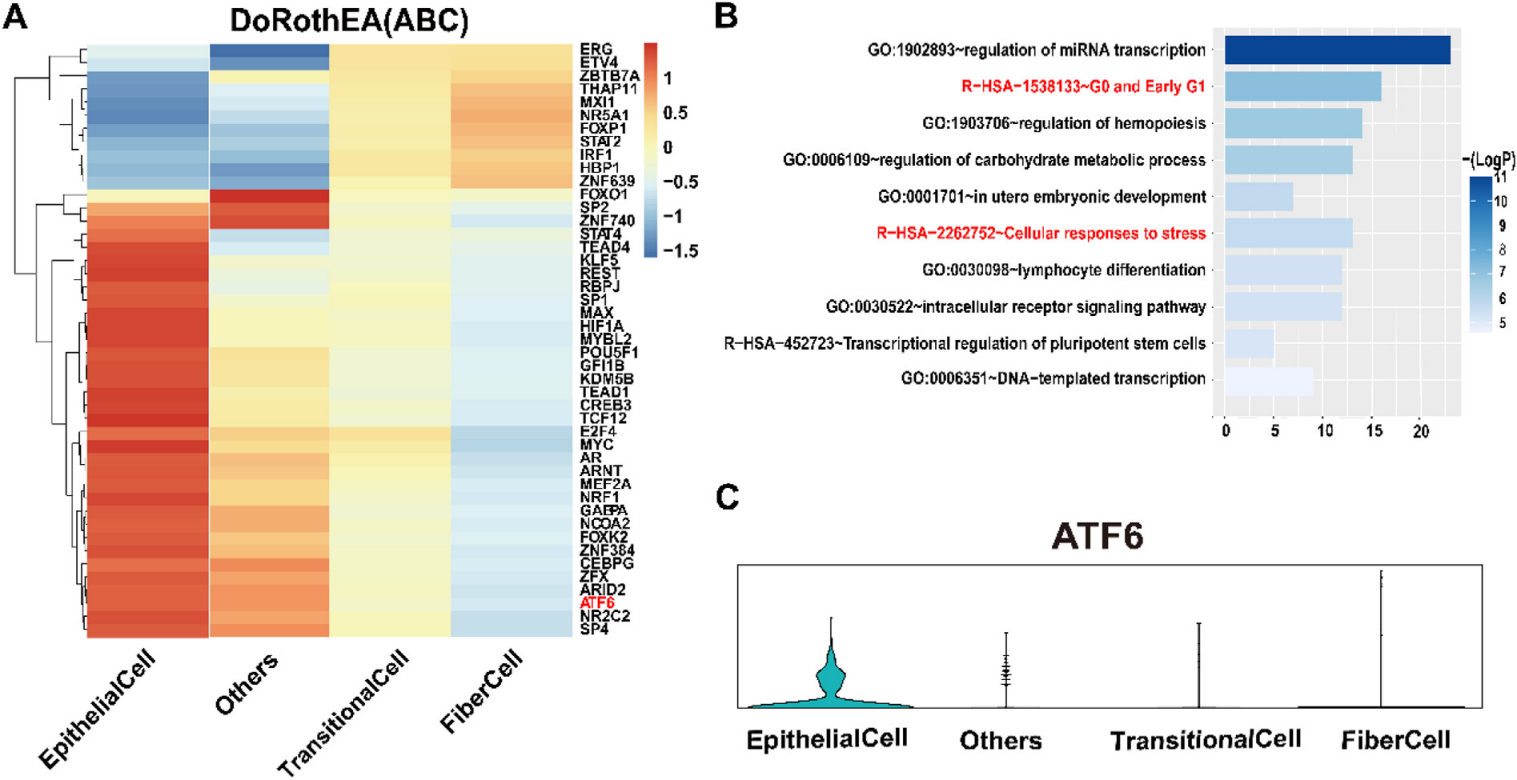
Transcription factor analysis. **(A)** Heatmap of top 45 highly variable transcription factor activities among the three severity groups. The *z*-scores of transcription factor activities are color-coded. **(B)** Predicted transcription factors were explored through gene ontology (GO) and Reactome databases for functional enrichment analysis using Metascape. **(C)** The expression distribution of ATF6 in each cluster. ATF6, activating transcription factor 6.

To further explore the regulatory mechanism of lens epithelial cells, we inferred transcription factor activity using the Dorothea algorithm and scored the activity of each regulon using the Viper inference tool.^29^, ^30^, ^31^ During the conversion of lens epithelial cells to fiber cells, the predicted expression patterns for related transcription factors exhibited two-fold: a gradual decrease from epithelial cells to fiber cells and a gradual increase from epithelial cells to fiber cells (Fig. 3A). Functional enrichment analyses of GO and REACTOME were performed using Metascape software.^32^ The enrichment analysis of biological processes within the GO revealed that the top 2 were GO: 1902893∼regulation of miRNA transcription and GO: 1903706∼regulation of hemopoiesis. Similarly, the enrichment analysis of the pathway within the REACTOME revealed that the top 2 were R-HSA-1538133∼GO and early G1 and R-HAS-2262752∼cellular responses to stress (Fig. 3B). It indicates that cell cycle and cell homeostasis are major factors for driving the differentiation of lens epithelial cells into fiber cells.

As we know, ATF6 is a pivotal transcription factor that orchestrates a complex network of cellular responses to maintain homeostasis. Its actions are critical for the cell's ability to adapt to changing conditions, making it a key player in cellular health and disease pathogenesis.^33^ In addition, prior research has established ATF6 as an important regulator in development and EMT.^34^^,^^35^ Furthermore, Clark et al found that during zebrafish development, ATF6 activity was the highest in the lens, skeletal muscle, fins, and gills.^36^ It suggested the crucial role of ATF6 in the lens. We then examined the expression patterns of ATF6 in the four lens clusters to pinpoint those specific to lens epithelial cells. Through this analysis, ATF6α was identified as being highly expressed within the epithelial cell clusters (Fig. 3C). The ATF6 target region is primarily determined through the following approach, which has been validated in other studies, for example, the analysis of the regulatory network of RNA binding protein-exon skipping events in the article titled “SexAnnoDB, a knowledgebase of sex-specific regulations from multi-omics data of human cancers”. We utilized the R software package “ArchR” to analyze scATAC-seq data initially. We integrated scRNA and scATAC data to identify and predict the chromatin open regions in lens epithelial cells and lens fiber cells. Furthermore, we utilized the ATF6 consistency sequence to traverse the open chromatin regions of the lens epithelial cells, capturing potential binding regions or genes of ATF6 in the cluster. Using this novel strategy, we identified 312 ATF6 target genes in lens epithelial cells (Table S3), including genes previously linked to lens development through multiple studies, such as PAX2 and MAF.^37^^,^^38^

These findings indicate that the active form of ATF6, ATF6α, is present and likely playing a role in the transcriptional regulation within these cells.

### Inhibition of ATF6 alleviates the mesenchymal transformation process of lens epithelial cells

**Figure 4:**
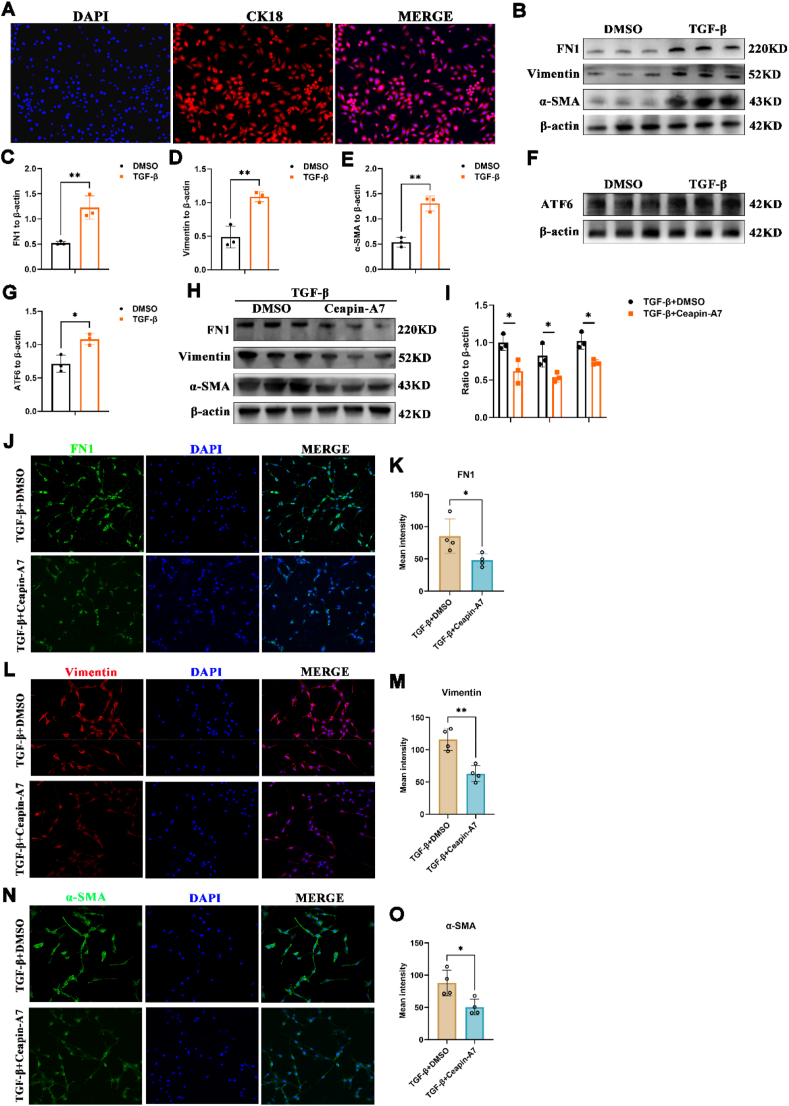
Increased ATF6 protein level in TGF-β1 treated primary lens epithelial cells. **(A)** Identification of mouse lens primary epithelial cells (DAPI, blue; CK18, red). **(B**–**E)** The protein levels and quantitative charts of FN1, vimentin, and α-SMA in TGF-β1 treated primary lens epithelial cells (*n* = 3/group; mean ± standard deviation; ∗∗*p* < 0.01; unpaired student's *t*-test). **(F, G)** Protein level and quantification of ATF6 in primary lens epithelial cells with TGF-β1 stimulation (*n* = 3/group; mean ± standard deviation; ∗*p* < 0.05; unpaired student's *t*-test). **(H, I)** Protein levels of FN1, vimentin, and α-SMA in TGF-β1 treated primary lens epithelial cells with DMSO or Ceapin-A7 (*n* = 3/group; mean ± standard deviation; ∗*p* < 0.05; unpaired student's *t*-test). **(J**–**O)** The immunofluorescence and quantitative intensity of FN1, vimentin, and α-SMA in the groups mentioned above (scale: 50 μm). ATF6, activating transcription factor 6; FN1, fibronectin 1; α-SMA, α-smooth muscle actin; TGF-β1, transforming growth factor beta 1.

The formation and progression of secondary cataracts are attributed to the mesenchymal transformation of lens epithelial cells.^39^ To investigate the role of ATF6 in EMT progression, we isolated primary lens epithelial cells from C57BL/6J mice. These cells were identified using the epithelial cell marker CK18^40^ (Fig. 4A). We then treated primary lens epithelial cells with TGF-β1 for 48 h. Western blot assays showed significant up-regulation of EMT markers in TGF-β1 treated primary lens epithelial cells, including fibronectin 1 (FN1), vimentin, and α-smooth muscle actin (α-SMA) (Fig. 4B–E). These findings confirmed the successful induction of an EMT model in the primary epithelial cells. Our subsequent Western blot evaluation of ATF6 in TGF-β1-stimulated primary epithelial cells uncovered a notable increase in the protein expression of ATF6 (Fig. 4F–G).

Ceapin-A7 selectively modulates the ATF6 signaling pathway with an IC50 of 0.59 μM.^41^ As per the guidelines, a concentration ten-fold higher is deemed to yield the optimal inhibitory efficiency. Consequently, in our study, SRA01/04 cells were treated with either DMAO or 6 μM Ceapin-A7 for a duration of 24 h prior to the establishment of an EMT model. Western blot analysis indicated a significant reduction in the protein levels of FN1, vimentin, and α-SMA in SRA01/04 cells following treatment with Ceapin-A7 (Fig. 4H, I). Additionally, immunofluorescence assays demonstrated the attenuation of epithelial interstitial fibrosis (Fig. 4J–O).

Additionally, the human lens epithelial cell line SRA01/04 was selected for our investigation because of its well-characterized and consistent behavior *in vitro*, as noted in previous studies.^42^ The results obtained from the SRA01/04 cell line were consistent with those observed in primary lens epithelial cells (Fig. S2). Taken together, our results show that ATF6 promotes the EMT process in lens epithelial cells, and antagonizing ATF6 effectively ameliorates epithelial interstitial fibrosis.

### Inhibition of ATF6 can alleviate epithelial-mesenchymal fibrosis by reducing STAT3 phosphorylation

**Figure 5:**
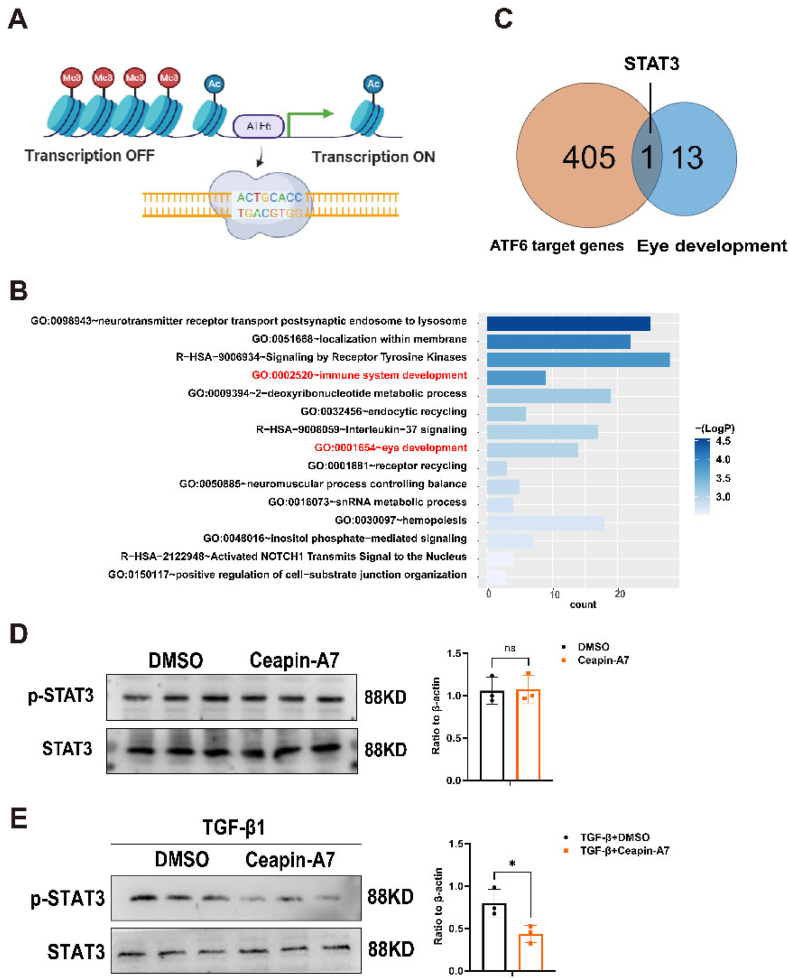
ATF6 function and target gene pathway analysis. **(A)** ATF6 binding schematic diagram, based on assays for ATAC-seq and using ATF6 motif for target gene prediction (BioRender: https://app.biorender.com). **(B)** Functional enrichment analysis for ATF6 target genes through gene ontology (GO) and Reactome databases. **(C)** STAT3 was the only gene located within 500 bp of the ATF6 promoter region. ATF6 promoter region TSS 500 gene overlays eye development pathways. **(D)** The protein level and quantification of STAT3 and p-STAT3 in primary lens epithelial cells treated with DMSO or Ceapin-A7 (*n* = 3/group; mean ± standard deviation; ∗∗*p* < 0.01; unpaired student's *t*-test). **(E)** The protein level and quantitative chart of STAT3 and p-STAT3 in TGF-β1 mediated primary lens epithelial cells treated with DMSO or Ceapin-A7 (*n* = 3/group; mean ± standard deviation; ∗∗*p* < 0.01; unpaired student's *t*-test). ATF6, activating transcription factor 6; TGF-β1, transforming growth factor beta 1; STAT3, signal transducer and activator of transcription 3; p-STAT3, phosphorylated STAT3.

We scanned the open chromatin regions of lens epithelial cells using the ATF6 motif sequence and predicted the downstream genes of ATF6 using the online software FIMO to delve into the regulatory mechanism of ATF6 in inhibiting fiber cells (Fig. 5A). Subsequently, GO biological function and Reactome biological pathway enrichment analyses were then performed using Metascape. The genes targeted by ATF6 in lens epithelial cells were found to be associated with the development of the immune system and the eye (Fig. 5B). Further analysis focused on downstream genes related to eye development, revealing STAT3 as the sole gene within 500 bp of the ATF6 promoter region (Fig. 5C). In primary lens epithelial cells treated with Ceapin-A7, no discernible changes were noted (Fig. 5D). However, upon subsequent activation with TGF-β1, a marked reduction in the protein levels of p-STAT3 was observed (Fig. 5E). Furthermore, in SRA01/04 cells that were both stimulated with TGF-β1 and treated with Ceapin-A7, a similar decrease in p-STAT3 protein levels was also noted (Fig. S3).

In conclusion, our study uncovered that inhibition of ATF6 could alleviate epithelial-mesenchymal fibrosis by reducing STAT3 phosphorylation.

## Discussion

Utilizing scRNA-seq analysis, we performed an extensive data investigation of lens cells from fetuses aged 9–23 weeks. Our analysis unveiled the existence of two additional cell groups, distinct from the epithelial cells and fibers of the lens, namely transitional cells and other cells. To validate our findings, we compared our data with previously published results of the snoRNA-seq nucleus-based adult lens. Remarkably, the differential genes identified in the literature exhibited similar expression patterns in our datasets as shown in Figure S1A and B. Within our datasets, three genes demonstrated high expression in lens epithelial cells. Among three, FOXE3, previously reported, was confirmed. The other two were SLC7A8 and SPARCL1. SLC7A8, recognized as a transporter protein, displayed heightened expression in lens epithelial cells and is associated with congenital or age-related cataracts.^43^ Conversely, SPARCL1, a secretory glycoprotein belonging to the SPARC stromal cell protein family, lacks known associations with the lens. While SPARCL1 is involved in cell adhesion, migration, and proliferation, its exact function in the lens is unclear. Our data highlights the elevated expression of SPARCL1 in lens epithelial cells, hinting at a potential impact within this cell population.

During embryonic eye development, the lens is primarily induced by fibroblast growth factors, bone morphogenetic proteins, and the Wnt transduction pathway.^44^ Smad-dependent bone morphogenetic protein receptor signaling can rearrange the actin cytoskeleton, inducing the inversion of the lens plate and promoting fetal lens development.^45^ Lens proteins produced by the lens fibers during this period serve as crucial structural components, with their unique spatial structure and abundant content playing a pivotal role in maintaining lens transparency. By employing DoRothEA, analysis software for the interaction between transcription factors and target genes, we conducted a transcription factor analysis on the lens subpopulation to identify the developmental regulatory transcription factors of lens epithelial cells and lens fiber cells during lens development. Our data indicate that the transcription factor FOXP1 (forkhead box P1) gradually increases its expression in lens epithelial cells, while MYC expression progressively decreases in lens fiber cells.^46^ These transcription factors have been associated with lens differentiation.^47^ The enrichment of transcription factor biological pathways is evident in the cell cycle and cell pressure. ATF6, a pivotal transcription factor in the endoplasmic reticulum stress response, is hypothesized to play a significant role in regulating lens cell homeostasis.

Numerous researchers have implicated ATF6 in maintaining the balance of lens cells.^48^, ^49^, ^50^ Notably, a 2015 study by Xu et al investigated a two-year-old with a biallelic loss-of-function mutation in ATF6 and observed only retinal changes, with no cataracts detected.^51^ However, the absence of cataracts in this single case does not definitively exclude ATF6's potential involvement in lens homeostasis. It is crucial to recognize the complexity of phenotypic variability due to genetic mutations. Compensatory mechanisms or genetic modifiers might mask the lens phenotype in certain cases. Furthermore, we must consider the potential for ATF6 to play differential roles across various ethnicities. This demographic difference could be significant, as genetic backgrounds can significantly influence the manifestation and impact of genetic mutations.^52^ In addition, our experiments have substantiated this hypothesis, confirming that ATF6 is a cataract-related transcription factor involved in the regulation of lens homeostasis.

ATAC-seq delineates four subpopulations of lens cell populations. To facilitate a comprehensive analysis of cell volume, we performed ATAC-seq analysis on 101 lens epithelial cells and 302 lens fiber cells. While an overarching analysis of all four subpopulations proved challenging, we specifically performed epigenetic and transcriptomic regulatory expression analysis for lens epithelial cells.

However, our study acknowledges certain limitations. The normal process of differentiation and EMT are two processes. The EMT process is intricately linked to the development of secondary cataracts. In our research, the single-cell data of the normal process of differentiation serves as a potential cue. Following the potential cue, we have conducted additional experiments under pathological conditions to delve deeper into our investigation. While this approach offers a distinct and valuable perspective on the early stages of lens development — a critical phase for comprehending the foundational biological processes that could be compromised in cataract formation — this data could not accurately reveal the underlying mechanisms of EMT. To address this, we intend to expand our investigation by acquiring scRNA-seq data from the lens tissues of individuals afflicted with secondary cataracts. This will enable us to delve deeper into the molecular mechanisms at play and potentially uncover novel insights into the progression of secondary cataracts.

ATF6 is triggered by endoplasmic reticulum stress, leading to its cleavage and subsequent nuclear translocation. However, it is unclear where the endoplasmic reticulum stress would arise in fetal development. To rectify this, we plan to conduct additional experiments that will focus on the expression and activation patterns of ATF6 across various stages of fetal development. These studies will provide a clearer picture of the temporal and spatial dynamics of ATF6's activity and its association with endoplasmic reticulum stress.

Mechanically, we identified STAT3 as the downstream target gene of ATF6 by integrating ATAC-seq and scRNA-seq. Western blot analysis also revealed the regulation of ATF6 on the protein level of p-STAT3. Nevertheless, we did not further explore whether ATF6 directly interacted with p-STAT3, warranting further investigation into this relationship. Although our findings imply the critical effect of ATF6 in epithelial cells, its role in animal models remains unclear. In the future, we aim to conduct a series of *in vivo* animal experiments to gain deeper insights.

In conclusion, we integrated scRNA-seq and scATAC-seq data to obtain two of the most important cell groups of the lens. Furthermore, we discovered cell group-specific markers and identified a transcription factor ATF6, which maintains epithelial homeostasis. Our experimental data indicated that ATF6 was significantly up-regulated in cellular models of cataract disease. Inhibiting ATF6 effectively alleviated the EMT process of lens epithelial cells. Our study provides a lens epithelial cell development atlas at the single-cell level, revealing that the overactivation of ATF6 may facilitate EMT in lens epithelial cells.

## CRediT authorship contribution statement

**Huiping Lu:** Writing – review & editing, Writing – original draft, Validation, Methodology, Data curation, Conceptualization. **Xianyang Liu:** Writing – review & editing, Writing – original draft, Validation, Methodology, Data curation. **Qian Zhou:** Writing – review & editing, Validation, Software, Methodology, Investigation. **Ruonan Li:** Software, Resources, Methodology. **Hangjia Zuo:** Software, Resources, Investigation. **Ke Hu:** Visualization, Software, Methodology. **Meng Tian:** Software, Investigation. **Hong Wang:** Software, Investigation. **Jianqiao Li:** Visualization, Supervision, Methodology. **Na Li:** Supervision, Resources, Investigation. **Shengping Hou:** Visualization, Supervision, Resources, Methodology, Funding acquisition.

## Funding

We gratefully acknowledge the financial support by the National Natural Science Foundation Project of China (No. 82271078), Youth Beijing Scholar (No. 076), Beijing Municipal Public Welfare Development and Reform Pilot Project for Medical Research Institutes (No. PWD&RPP-MRI, JYY2023-6).

## Conflict of interests

All authors declared no competing interests.
