# Supplementary material for: Integrated analysis of single-cell RNA-seq and ATAC-seq in lens epithelial cells: Unveiling the role of ATF6 as a key transcription factor
Source: Genes Dis. 2025 Mar 22;12(6):101610. doi: 10.1016/j.gendis.2025.101610 (PMC12361989; doi:10.1016/j.gendis.2025.101610)

Supplementary Table.1. Detailed information of samples in scRNA-seq.
Supplementary Table.2. Detailed information of samples in scATAC-seq.
Supplementary Table.3. The target genes and open chromatin regions.
Supplementary Fig. 1. (A) Proportion of epithelial cells and lens fibroblasts. (B) Single-nucleus expression profiles. (C) Comparative analysis of crucial transcription factor data obtained from lens epithelial cells of adults, zebrafish, and chickens.
Supplementary Fig. 2. Increased ATF6 protein level in TGF-β1 treated SRA01/04 cells. (A-D). The protein levels and quantitative charts of FN1, Vimentin, and α-SMA in TGF-β1 treated SRA01/04 cells (n=3/group; mean± SD; *P<0.05; **P<0.01; unpaired Student's t-test). (E, G). ATF6 protein level and quantification (n=3/group; mean± SD; *P<0.05; unpaired Student's t-test). (F, H-J). The protein levels of FN1, Vimentin, and α-SMA in TGF-β1 treated SRA01/04 cells with DMSO or Ceapin-A7 (n=3/group; mean± SD; *P<0.05; ***P<0.001; unpaired Student's t-test). (K-M). The immunofluorescence of FN1, Vimentin, and α-SMA respectively (scar: 100 μm). **Supplementary Fig. 3.** The protein level and quantification of STAT3 and P-STAT3 in TGF-β1 mediated SRA01/04 cells treated with DMSO or Ceapin-A7 (n=3/group; mean± SD; **P<0.01; unpaired Student's t-test).

Supplementary Table.1. Detailed information of samples in scRNA-seq.

Sample ID	Number of cells	Mean reads per cell	Median genes per cell	Sample Type	Sample origin
PCW9	15,831	25,368	2,018	Whole eyeball	China
PCW10	9,024	77,612	3,235	Whole eyeball	China
PCW11	6,535	43,909	2,560	Whole eyeball	China
PCW14	9,913	66,003	2,510	Whole eyeball	China
PCW15	9,532	30,383	1,664	Whole eyeball	China
PCW18	10,061	55,903	2,153	Whole eyeball	China
PCW20	11,910	37,180	2,086	Whole eyeball	China
PCW23	6,305	56,070	1,717	Whole eyeball	China

Supplementary Table.2. Detailed information of samples in scATAC-seq.

Sample ID	Number of cells	Sample Type	Sample origin
PW9	3397	Whole eyeball	China
PW10	4826	Whole eyeball	China
PW11	6238	Whole eyeball	China
PW11-rep	14494	Whole eyeball	China
PW14	4505	Whole eyeball	China
PW15	11326	Whole eyeball	China
PW18	10131	Whole eyeball	China
PW19	2923	Whole eyeball	China
PW20	7262	Whole eyeball	China
PW23	3912	Whole eyeball	China

Supplementary Table 3. The target genes and open chromatin regions

ID	Start	End	Score	Strand	Description
SMIM4::chr3:52524750-52525250	246	259	69.6	-	Name=MA1466.1_SMIM4::chr3:52524750-52525250-;Alias=ATF6;ID=MA1466.1-ATF6-1-SMIM4::chr3:52524750-52525250;pvalue=1.1e-07;qvalue=0.695;sequence=CGCTGACGTGGCAG;
AHRR::chr5:359387-359887	80	93	42.9	+	Name=MA1466.1_AHRR::chr5:359387-359887+;Alias=ATF6;ID=MA1466.1-ATF6-1-AHRR::chr5:359387-359887;pvalue=5.1e-05;qvalue=1;sequence=TGCTGACGGGGCAC;
RBM19::chr12:113945599-113946099	279	292	40	-	Name=MA1466.1_RBM19::chr12:113945599-113946099-;Alias=ATF6;ID=MA1466.1-ATF6-1-RBM19::chr12:113945599-113946099;pvalue=9.95e-05;qvalue=1;sequence=GAATGCCGTGGCCG;
GABRA5::chr15:26555947-26556447	440	453	40.5	-	Name=MA1466.1_GABRA5::chr15:26555947-26556447-;Alias=ATF6;ID=MA1466.1-ATF6-1-GABRA5::chr15:26555947-26556447;pvalue=9e-05;qvalue=1;sequence=TCTTCACGTGGCAA;
GPR139::chr16:20120381-20120881	15	28	44.4	-	Name=MA1466.1_GPR139::chr16:20120381-20120881-;Alias=ATF6;ID=MA1466.1-ATF6-1-GPR139::chr16:20120381-20120881;pvalue=3.62e-05;qvalue=1;sequence=TAATGGCGTGGAAA;
SNORA37::chr18:54173184-54173684	272	285	40.9	+	Name=MA1466.1_SNORA37::chr18:54173184-54173684+;Alias=ATF6;ID=MA1466.1-ATF6-1-SNORA37::chr18:54173184-54173684;pvalue=8.12e-05;qvalue=1;sequence=GTATGACGCAGCAG;
BRK1::chr3:10115177-10115677	385	398	49.3	+	Name=MA1466.1_BRK1::chr3:10115177-10115677+;Alias=ATF6;ID=MA1466.1-ATF6-1-BRK1::chr3:10115177-10115677;pvalue=1.16e-05;qvalue=1;sequence=ACGTGACGTTGCAC;
KIF7::chr15:89648207-89648707	355	368	47.9	+	Name=MA1466.1_KIF7::chr15:89648207-89648707+;Alias=ATF6;ID=MA1466.1-ATF6-1-KIF7::chr15:89648207-89648707;pvalue=1.6e-05;qvalue=1;sequence=CGGTGCCGTGGCGG;
MIR3123::chr1:241072000-241072500	257	270	42.1	+	Name=MA1466.1_MIR3123::chr1:241072000-241072500+;Alias=ATF6;ID=MA1466.1-ATF6-1-MIR3123::chr1:241072000-241072500;pvalue=6.16e-05;qvalue=1;sequence=ctgtggcgttgcat;
FZD5::chr2:207767436-207767936	4	17	43.6	+	Name=MA1466.1_FZD5::chr2:207767436-207767936+;Alias=ATF6;ID=MA1466.1-ATF6-1-FZD5::chr2:207767436-207767936;pvalue=4.32e-05;qvalue=1;sequence=TGATGACGCTGCGG;
DNER::chr2:229676906-229677406	235	248	41.8	-	Name=MA1466.1_DNER::chr2:229676906-229677406-;Alias=ATF6;ID=MA1466.1-ATF6-1-DNER::chr2:229676906-229677406;pvalue=6.64e-05;qvalue=1;sequence=GGCTGATGTGTCAA;
PACRGL::chr4:20744629-20745129	38	51	43.4	+	Name=MA1466.1_PACRGL::chr4:20744629-20745129+;Alias=ATF6;ID=MA1466.1-ATF6-1-PACRGL::chr4:20744629-20745129;pvalue=4.56e-05;qvalue=1;sequence=aaatgacgtgttaa;
ZNF800::chr7:127398193-127398693	257	270	48.2	+	Name=MA1466.1_ZNF800::chr7:127398193-127398693+;Alias=ATF6;ID=MA1466.1-ATF6-1-ZNF800::chr7:127398193-127398693;pvalue=1.52e-05;qvalue=1;sequence=TTATGATGTGGCAT;
PAXIP1-AS2::chr7:154811113-154811613	377	390	44.1	+	Name=MA1466.1_PAXIP1-AS2::chr7:154811113-154811613+;Alias=ATF6;ID=MA1466.1-ATF6-1-PAXIP1-AS2::chr7:154811113-154811613;pvalue=3.85e-05;qvalue=1;sequence=TGATGAGGTGGCAT;
LOC100506585::chr7:157756400-157756900	456	469	40.6	-	Name=MA1466.1_LOC100506585::chr7:157756400-157756900-;Alias=ATF6;ID=MA1466.1-ATF6-1-LOC100506585::chr7:157756400-157756900;pvalue=8.62e-05;qvalue=1;sequence=TAATGACATGGAAA;
MYPN::chr10:68155939-68156439	154	167	40.1	-	Name=MA1466.1_MYPN::chr10:68155939-68156439-;Alias=ATF6;ID=MA1466.1-ATF6-1-MYPN::chr10:68155939-68156439;pvalue=9.82e-05;qvalue=1;sequence=CTCTGACATGGCCT;
FGF6::chr12:4444786-4445286	293	306	42.3	+	Name=MA1466.1_FGF6::chr12:4444786-4445286+;Alias=ATF6;ID=MA1466.1-ATF6-1-FGF6::chr12:4444786-4445286;pvalue=5.86e-05;qvalue=1;sequence=GGGTCACGTGGAAT;
C16orf78::chr16:49581872-49582372	21	34	49	-	Name=MA1466.1_C16orf78::chr16:49581872-49582372-;Alias=ATF6;ID=MA1466.1-ATF6-1-C16orf78::chr16:49581872-49582372;pvalue=1.24e-05;qvalue=1;sequence=TGGTGACATGTCAG;
C16orf78::chr16:49581872-49582372	19	32	42.9	+	Name=MA1466.1_C16orf78::chr16:49581872-49582372+;Alias=ATF6;ID=MA1466.1-ATF6-2-C16orf78::chr16:49581872-49582372;pvalue=5.08e-05;qvalue=1;sequence=AGCTGACATGTCAC;
PREX1::chr20:48818870-48819370	247	260	59.3	-	Name=MA1466.1_PREX1::chr20:48818870-48819370-;Alias=ATF6;ID=MA1466.1-ATF6-1-PREX1::chr20:48818870-48819370;pvalue=1.17e-06;qvalue=0.955;sequence=TGGTGACGTGGAAA;

MIR5739::chr22:28527660-28528160	260	273	40.8	+	Name=MA1466.1_MIR5739::chr22:28527660-28528160+;Alias=ATF6;ID=MA1466.1-ATF6-1-MIR5739::chr22:28527660-28528160;pvalue=8.23e-05;qvalue=1;sequence=CCATGACATAgcag;
MIR4330::chrX:151079850-151080350	34	47	43.2	-	Name=MA1466.1_MIR4330::chrX:151079850-151080350-;Alias=ATF6;ID=MA1466.1-ATF6-1-MIR4330::chrX:151079850-151080350;pvalue=4.82e-05;qvalue=1;sequence=AGATGACATGGCTC;
FNIP1::chr5:131796984-131797484	199	212	49.2	+	Name=MA1466.1_FNIP1::chr5:131796984-131797484+;Alias=ATF6;ID=MA1466.1-ATF6-1-FNIP1::chr5:131796984-131797484;pvalue=1.21e-05;qvalue=1;sequence=CCGTCACGTGGCAG;
FNIP1::chr5:131796984-131797484	118	131	45.8	+	Name=MA1466.1_FNIP1::chr5:131796984-131797484+;Alias=ATF6;ID=MA1466.1-ATF6-2-FNIP1::chr5:131796984-131797484;pvalue=2.63e-05;qvalue=1;sequence=GCATCACGTGGCGG;
TFEC::chr7:115763449-115763949	225	238	43.5	+	Name=MA1466.1_TFEC::chr7:115763449-115763949+;Alias=ATF6;ID=MA1466.1-ATF6-1-TFEC::chr7:115763449-115763949;pvalue=4.49e-05;qvalue=1;sequence=CAATGACGTAGAAA;
AOC1::chr7:150857703-150858203	252	265	41.9	-	Name=MA1466.1_AOC1::chr7:150857703-150858203-;Alias=ATF6;ID=MA1466.1-ATF6-1-AOC1::chr7:150857703-150858203;pvalue=6.42e-05;qvalue=1;sequence=GAGTGCCGTGGCGC;
MAF::chr16:78930564-78931064	201	214	40.4	-	Name=MA1466.1_MAF::chr16:78930564-78931064-;Alias=ATF6;ID=MA1466.1-ATF6-1-MAF::chr16:78930564-78931064;pvalue=9.04e-05;qvalue=1;sequence=AGGTGCCGTGGAAC;
PAX2::chr10:100782397-100782897	210	223	42.4	+	Name=MA1466.1_PAX2::chr10:100782397-100782897+;Alias=ATF6;ID=MA1466.1-ATF6-1-PAX2::chr10:100782397-100782897;pvalue=5.72e-05;qvalue=1;sequence=TGATGACCTGGCGC;
…					
OR51M1::chr11:5402555-5403055	185	198	44.7	-	Name=MA1466.1_OR51M1::chr11:5402555-5403055-;Alias=ATF6;ID=MA1466.1-ATF6-1-OR51M1::chr11:5402555-5403055;pvalue=3.36e-05;qvalue=1;sequence=TGATGACGTGAAGA;
LOC101928557::chr16:85780261-85780761	58	71	41.9	+	Name=MA1466.1_LOC101928557::chr16:85780261-85780761+;Alias=ATF6;ID=MA1466.1-ATF6-1-LOC101928557::chr16:85780261-85780761;pvalue=6.39e-05;qvalue=1;sequence=CTCTCACGTGGCCG;
YJU2::chr19:4259507-4260007	333	346	50.4	-	Name=MA1466.1_YJU2::chr19:4259507-4260007-;Alias=ATF6;ID=MA1466.1-ATF6-1-YJU2::chr19:4259507-4260007;pvalue=9.14e-06;qvalue=1;sequence=CACTGACGTGGAAC;
YJU2::chr19:4259507-4260007	15	28	41.8	-	Name=MA1466.1_YJU2::chr19:4259507-4260007-;Alias=ATF6;ID=MA1466.1-ATF6-2-YJU2::chr19:4259507-4260007;pvalue=6.58e-05;qvalue=1;sequence=TGGTGATGTGTCCC;
NFATC2::chr20:51508034-51508534	220	233	40.1	-	Name=MA1466.1_NFATC2::chr20:51508034-51508534-;Alias=ATF6;ID=MA1466.1-ATF6-1-NFATC2::chr20:51508034-51508534;pvalue=9.82e-05;qvalue=1;sequence=AGGTGGCGTGGTGA;
CPNE5::chr6:36839752-36840252	414	427	42.3	+	Name=MA1466.1_CPNE5::chr6:36839752-36840252+;Alias=ATF6;ID=MA1466.1-ATF6-1-CPNE5::chr6:36839752-36840252;pvalue=5.86e-05;qvalue=1;sequence=TGGGGACGTGGCGG;
ZDHHC2::chr8:17155952-17156452	442	455	50.8	-	Name=MA1466.1_ZDHHC2::chr8:17155952-17156452-;Alias=ATF6;ID=MA1466.1-ATF6-1-ZDHHC2::chr8:17155952-17156452;pvalue=8.23e-06;qvalue=1;sequence=CCCTGGCGTGGCAG;
PLPP5::chr8:38269040-38269540	186	199	57.4	+	Name=MA1466.1_PLPP5::chr8:38269040-38269540+;Alias=ATF6;ID=MA1466.1-ATF6-1-PLPP5::chr8:38269040-38269540;pvalue=1.81e-06;qvalue=1;sequence=CGCTGACGTGGCCA;
MIR4312::chr15:68789866-68790366	238	251	40.7	+	Name=MA1466.1_MIR4312::chr15:68789866-68790366+;Alias=ATF6;ID=MA1466.1-ATF6-1-MIR4312::chr15:68789866-68790366;pvalue=8.54e-05;qvalue=1;sequence=GAGTCACGTGGCCC;

Supplementary Fig. 1

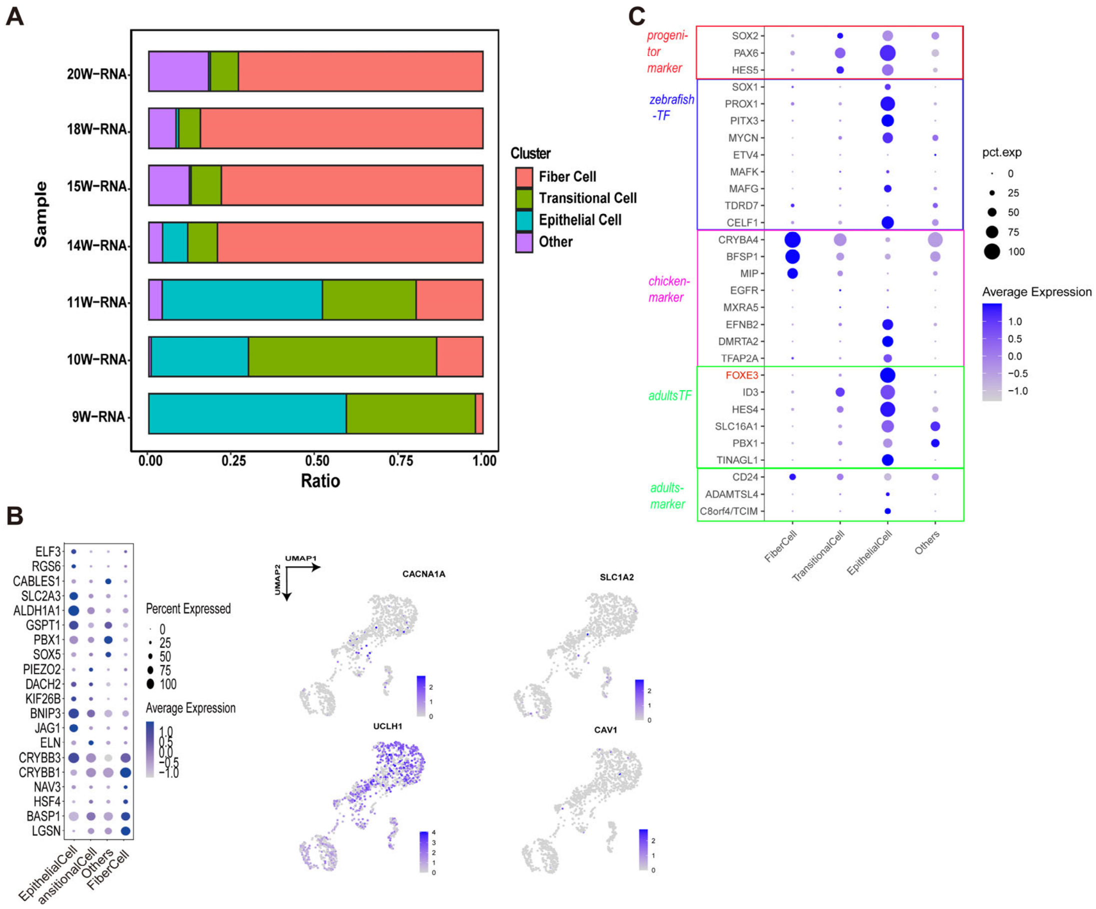

Supplementary Fig. 2

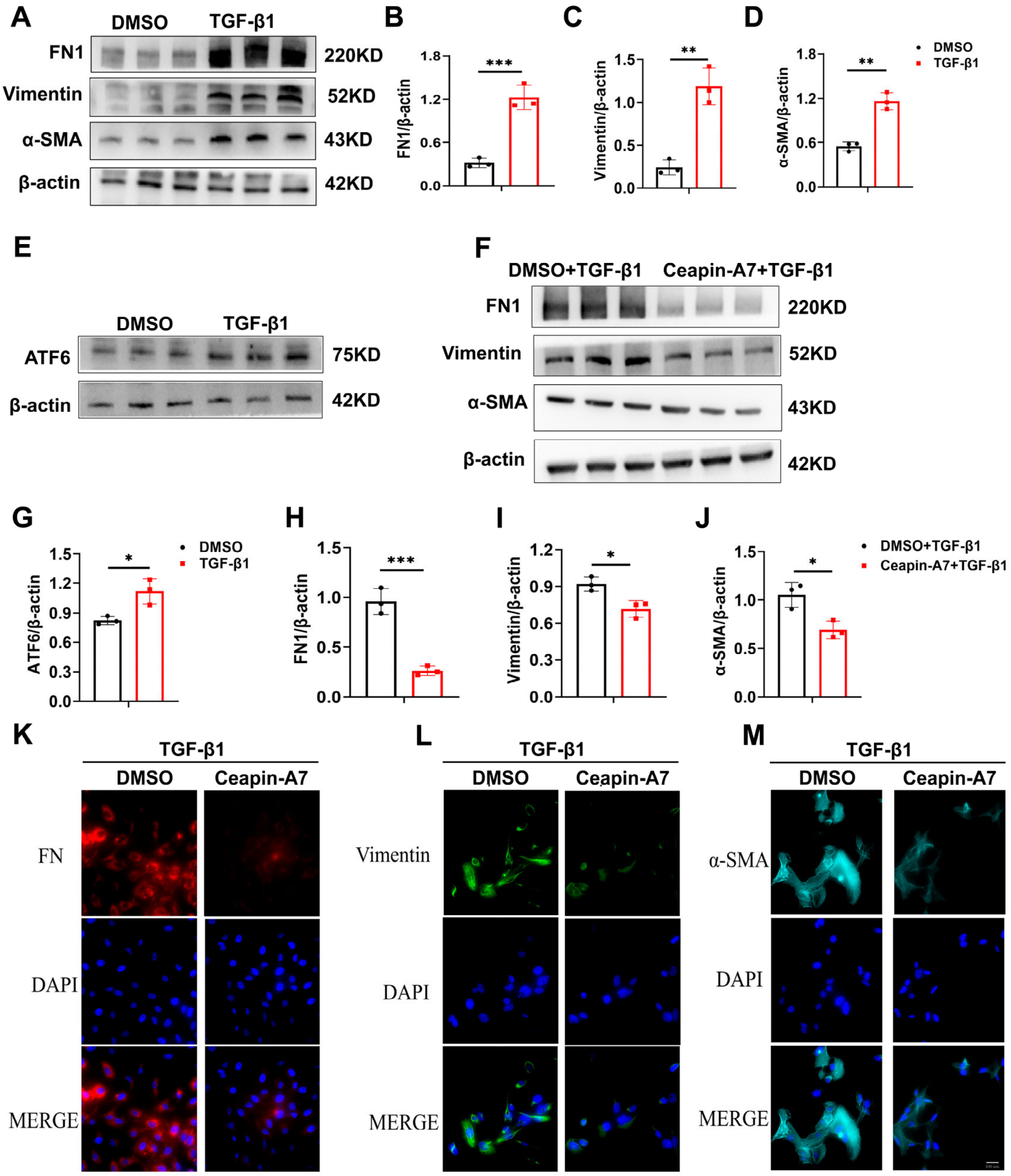

Supplementary Fig. 3

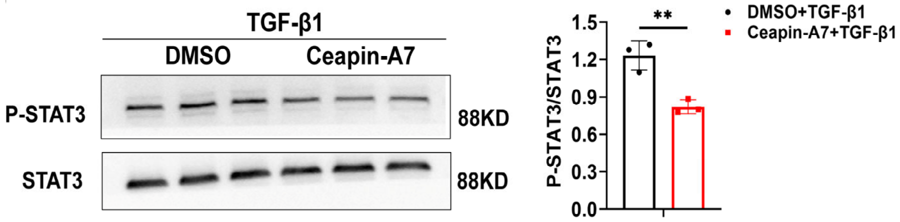

Supplement: Multimedia component 1 [file mmc1.pdf]
